# AMPK activation attenuates central sensitization in a recurrent nitroglycerin-induced chronic migraine mouse model by promoting microglial M2-type polarization

**DOI:** 10.1186/s10194-024-01739-w

**Published:** 2024-03-08

**Authors:** Guangshuang Lu, Shaobo Xiao, Fanchao Meng, Leyi Zhang, Yan Chang, Jinjing Zhao, Nan Gao, Wenjie Su, Xinghao Guo, Yingyuan Liu, Chenhao Li, Wenjing Tang, Liping Zou, Shengyuan Yu, Ruozhuo Liu

**Affiliations:** 1 Medical School of Chinese PLA, Beijing, 100853 China; 2 Department of Neurology, International Headache Center, The First Medical Center of Chinese PLA General Hospital, Fuxing Road 28, Haidian District, Beijing, 100853 China; 3 Department of Pediatrics, The Lu’an Hospital Affiliated to Anhui Medical University, The Lu’an People’s Hospital, Lu’an, China; 4 School of Medicine, Nankai University, Tianjin, 300071 China; 5 Department of Pediatrics, The First Medical Center of Chinese PLA General Hospital, Fuxing Road 28, Haidian District, Beijing, 100853 China

**Keywords:** Chronic migraine, AMP-activated protein kinase, Central sensitization, Trigeminal nucleus caudalis, Microglia

## Abstract

**Background:**

Energy metabolism disorders and neurogenic inflammation play important roles in the central sensitization to chronic migraine (CM). AMP-activated protein kinase (AMPK) is an intracellular energy sensor, and its activation regulates inflammation and reduces neuropathic pain. However, studies on the involvement of AMPK in the regulation of CM are currently lacking. Therefore, this study aimed to explore the mechanism underlying the involvement of AMPK in the central sensitization to CM.

**Methods:**

Mice with recurrent nitroglycerin (NTG)-induced CM were used to detect the expression of AMPK protein in the trigeminal nucleus caudalis (TNC). Following intraperitoneal injection of the AMPK activator 5-aminoimidazole-4-carboxyamide ribonucleoside (AICAR) and inhibitor compound C, the mechanical pain threshold, activity level, and pain-like behaviors in the mice were measured. The expression of calcitonin gene-related peptide (CGRP) and cytokines, M1/M2 microglia, and NF-κB pathway activation were detected after the intervention.

**Results:**

Repeated NTG injections resulted in a gradual decrease in AMPK protein expression, and the negative regulation of AMPK by increased ubiquitin-like plant homeodomain and RING finger domain 1 (UHRF1) expression may counteract AMPK activation by increasing ADP/ATP. AICAR can reduce the hyperalgesia and pain-like behaviors of CM mice, improve the activity of mice, reduce the expression of CGRP, IL-1β, IL-6, and TNF-α in the TNC region, and increase the expression of IL-4 and IL-10. Moreover, AMPK in TNC was mainly located in microglia. AICAR could reduce the expression of inducible NO synthase (iNOS) in M1 microglia and increase the expression of Arginase 1 (Arg1) in M2 microglia by inhibiting the activation of NF-κB pathway.

**Conclusions:**

AMPK was involved in the central sensitization of CM, and the activation of AMPK reduced neuroinflammation in NTG-induced CM mice. AMPK may provide new insights into interventions for energy metabolism disorders and neurogenic inflammation in migraine.

**Supplementary Information:**

The online version contains supplementary material available at 10.1186/s10194-024-01739-w.

## Introduction

Migraine is a prevalent neurological disorder characterized by recurrent headache attacks. According to the 2019 Global Burden of Disease Study (GBD), approximately 8–15% of individuals with migraine experience at least one attack per year [1]. This condition leads to a higher number of years lived with disability (YLDs) compared to all other neurological disorders combined [2, 3]. Migraine can be categorized into two types: episodic migraine (EM) and chronic migraine (CM). CM is diagnosed in patients who suffer from at least 15 headache days for more than three months, with at least eight of those days being related to migraines, as defined by the International Classification of Headache Disorders, 3rd edition (ICHD-III) [4]. It has been estimated that up to 5% of EM patients may progress to CM [4]. The severity of CM is significantly higher than that of EM, with up to 45% of patients seeking treatment at headache clinics experiencing daily or near-daily headaches [5]. This has a profound impact on the patient's overall quality of life.

The pathophysiology of CM is believed to involve central sensitization of the trigeminal vascular system and neuroinflammation [6, 7]. This is characterized by increased excitability and synaptic plasticity of sensory neurons, primarily occurring in the trigeminal nucleus caudalis (TNC) and thalamus. Neurogenic neuroinflammation in migraines refers to the inflammatory reactions in the central and peripheral components of the trigeminovascular system in response to neuronal activity [7]. Various factors, including microglia, astrocytes, inflammatory cytokines, neurotransmitters and receptors, and ion channels, contribute to the central sensitization of neurons. Microglia play a crucial role in this process and can exhibit different phenotypes upon activation. The M1 phenotype is associated with the release of pro-inflammatory cytokines such as IL-1β, IL-6, TNF-α, CCL2, reactive oxygen species (ROS), and inducible NO synthase (iNOS). On the other hand, the M2 phenotype can produce anti-inflammatory and immunosuppressive factors, including Arginase 1 (Arg1), Ym1, CD204, CD206, and IL-10 [8–10]. The production of proinflammatory molecules by microglia and their release near neuronal cell bodies are key factors in maintaining immune homeostasis in the central nervous system [11]. Changes in the levels of inflammatory cytokines, such as IL-1β, IL-6, IL-10, and TNF-α, have been observed in both the plasma and serum of patients with migraines and animal models [12–14].

Furthermore, abnormalities in energy metabolism have been reported in migraines, as evidenced by neuroimaging, biochemical, genetic, and therapeutic studies. Neuroimaging techniques such as ^31^P-MRS, ^1^H-MRS, and ^18^F-FDG-PET have revealed impaired oxidative phosphorylation of brain mitochondria in patients with migraines between attacks. This is characterized by increased ADP levels, decreased ATP levels, and elevated brain lactic acid levels, indicating a reduced energy reserve in migraine patients [15–21]. Additionally, many triggers or factors that worsen migraines are associated with energy metabolism and oxidative stress [22]. For instance, fasting, hunger, disrupted sleep, fatigue, and altered brain energy metabolism are directly linked to migraines. Intense exercise, chronic stress, weather and environmental changes, and strong sensory stimuli such as strong odors, bright lights, and loud noises can increase the demand for brain energy and availability, consequently elevating oxidative stress levels in the central nervous system.

The exact mechanism linking energy metabolism disorders and neurogenic inflammation in CM is currently not fully understood. However, AMP-activated protein kinase (AMPK) is a crucial intracellular energy sensor that detects levels of adenosine nucleotides [23]. When the intracellular AMP/ADP or ADP/ATP ratio increases, AMPK is activated and plays a role in various signaling pathways involved in regulating inflammation, autophagy, and mitochondrial homeostasis. This activation helps restore brain energy homeostasis. The negative regulators of AMPK are not well-known, but one such regulator is ubiquitin-like with plant homeodomain and RING finger domains 1 (UHRF1). UHRF1 inhibits AMPK through dephosphorylation under both basal and stress-induced activation conditions. Additionally, UHRF1 can promote fatty acid and protein synthesis by inhibiting AMPK [24].

AMPK has been shown to play a role in attenuating neuroinflammatory pain by inhibiting NF-κB activation and IL-1β expression [25]. However, there is a lack of studies investigating the specific mechanism by which AMPK is involved in regulating CM. This study proposes that the abnormal energy metabolism observed in migraines can activate the AMPK energy sensor and disrupt its regulation during the chronic phase of the condition. The objective of this study is to investigate the mechanism through which AMPK reduces neuroinflammation and contributes to the central sensitization observed in migraines.

## Methods

### Animals and ethics statement

The experiments were conducted using adult male C57BL/6J mice weighing between 20–30 g (SPF Biotechnology Co., Ltd., Beijing, China). The mice were individually housed in cages under specific conditions, including a room temperature of 23 ± 2 °C, humidity of 50 ± 10%, a 12-h light–dark cycle, and provided with ad libitum access to food and water. Following one week of adaptation to the laboratory environment, the mice were randomly assigned to different experimental groups. All experimental procedures were approved by the Institutional Animal Care and Use Committee of the Chinese PLA General Hospital and were conducted in accordance with the Regulations for the Administration of Affairs Concerning Experimental Animals.

### Animal modeling and experimental design

In this study, a mouse model of CM was induced by repeated injections of nitroglycerin (NTG). NTG was obtained at a concentration of 5 mg/mL in a solution containing 30% alcohol, 30% propylene glycol, and water (Beijing Reagent, China). Prior to administration, NTG was freshly diluted in 0.9% saline (normal saline, NS) to a concentration of 1 mg/mL. The mice were intraperitoneally (i.p.) injected with either 10 mg/kg NTG or an equal volume of vehicle (NS) every second day for a total of 9 days (five injections in total).

The experiment was conducted in two steps.

**Fig. 1:**
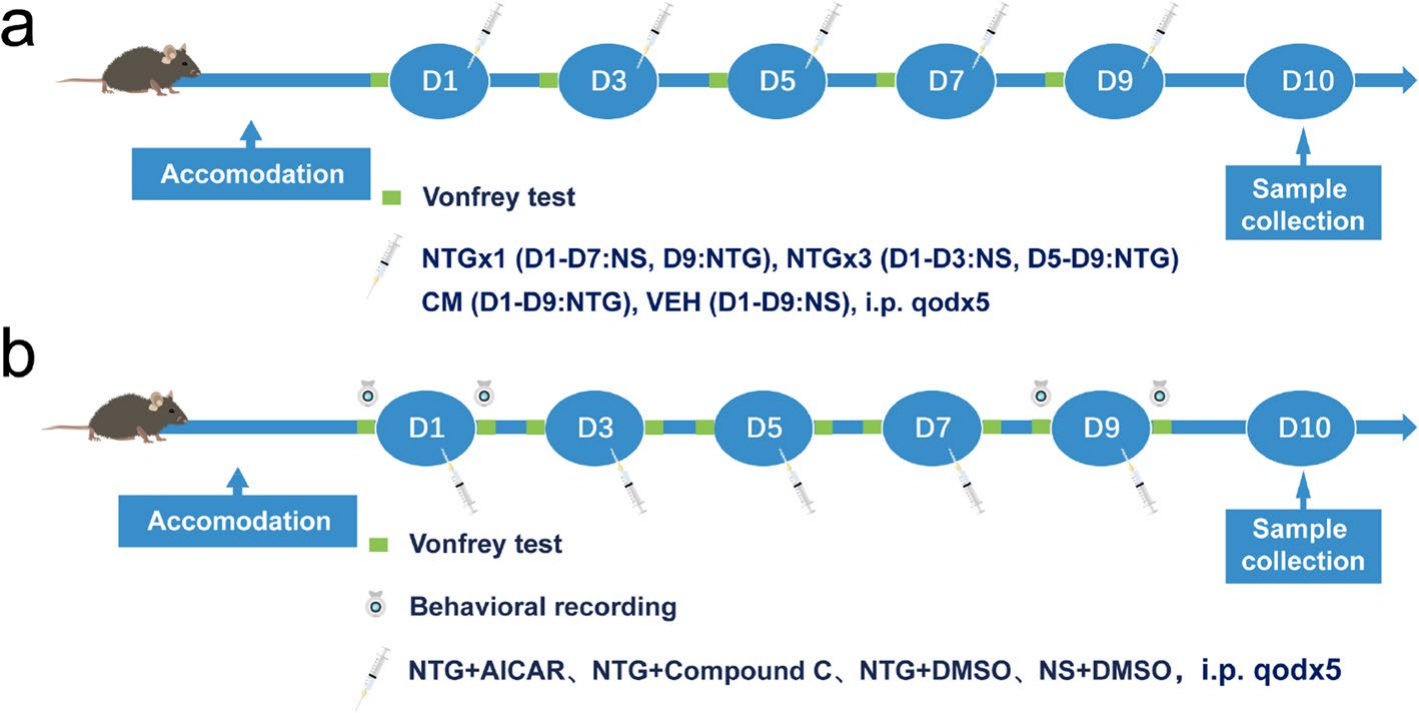
Experimental flow chart. **a** Experiment design for studying the changes in AMP-activated protein kinase (AMPK) activity in the chronic migraine (CM) model mice during the modeling process. **b** Experimental design to explore the mechanism of AMPK regulating neuroinflammation in CM mice. NTG: nitroglycerin, NS: 0.9% saline, AICAR: AMPK activator 5-aminoimidazole-4-carboxamide1-β-D-ribofuranoside, Compound C: AMPK inhibitor, DMSO: solvent dimethyl sulfoxide, D: day

Firstly, we investigated the changes in AMPK activity in the CM model mice during the modeling process. The C57BL/6 mice were randomly divided into four groups: NTGx1, NTGx3, CM, and VEH (control). Each group consisted of 8–12 mice. To ensure consistency in the injection procedure, all mice received a total of five injections, either NTG or an equivalent amount of NS. Behavioral tests were conducted before the injections in the CM and VEH groups. On the 10th day, we collected the TNC tissues from the mice in each group (Fig. 1a).

In the next step, we aimed to intervene with AMPK and investigate the mechanism by which AMPK regulates neuroinflammation in CM mice. Two compounds were utilized in the process, namely the AMPK activator 5-aminoimidazole-4-carboxamide-1-β-D-ribofuranoside (AICAR, ab120358, Abcam) and the AMPK inhibitor compound C (Abcam, ab120843). AICAR has been extensively studied as an AMPK activator in various cell types. It mimics the function of AMP to activate AMPK [26, 27]. Both AICAR and compound C have been used in neurological disease studies via gavage or intraperitoneal injection [28–34]. Based on previous literature, the researchers categorized the AICAR doses as 500 μg/g, 250 μg/g, and 125 μg/g [35–37], while the compound C doses were categorized as 20 μg/g, 10 μg/g, and 5 μg/g [32, 38]. AICAR was dissolved in 10% dimethyl sulfoxide (DMSO) to prepare a concentration of 25 μg/μL in phosphate-buffered saline (PBS, 0.1 M). Compound C was prepared at a concentration of 1 μg/μL in PBS. Both AICAR and compound C were administered intraperitoneally (i.p.) 5 min after the injection of NTG or 0.9% NS. After one week of acclimatization, the mice were randomly divided into four groups: NTG + AICAR, NTG + Compound C, NTG + DMSO, and NS + DMSO. Behavioral tests were conducted before and 2 h after each injection. On the 10th day, we collected the TNC tissues from the mice in each group (Fig. 1b).

### Behavioral tests

#### Cutaneous allodynia behavior

To evaluate the cutaneous allodynia behavior in the mice, mechanical thresholds were measured in the periorbital region and hindpaw plantar region every other day before or 2 h after NTG injections in different parts of experiment. The mice were habituated to a Plexiglas box with a mesh grid bottom for 15–20 min before the measurement. Mechanical thresholds were assessed by applying von Frey filaments (vFF) perpendicularly to the periorbital region and hindpaw plantar. The filaments were bent for approximately 3 s or until a positive response was evoked. The vFF was calibrated according to the manufacturer’s instructions. The force range of the vFF applied to the periorbital and hindpaw plantar regions was 0.008–0.4 g and 0.07–4 g, respectively. A positive response in the periorbital region was defined as shaking, repeated pawing, or cowering away from the filament. In the hindpaw, the responses included lifting, shaking, or licking of the paw after stimulation. The filaments were applied sequentially to determine the mechanical thresholds. If there was no response, a heavier filament was used, and if there was a response, a lighter filament was tested. The mechanical thresholds were defined as the force that first evoked a positive response three times in five or more trials. To minimize bias, the researchers conducted the mechanical threshold tests at the same time of the day (between 8:00 and 18:00) and were blinded to the treatment groups during testing.

#### Migraine-like behavioral recording

The experiment was conducted in a quiet room with an illumination of 30 lx. The experiment took place in a 30 × 40 cm box. Prior to the NTG injection, the mice were brought into the test room 3 h in advance for acclimatization. The mice were placed in the box for 10 min as a baseline measurement of activity and then again 2 h after the NTG injection to observe post-treatment responses. The decision to monitor behavior at the 2-h mark was based on previous studies that indicated the lowest value of mechanical pain area in mice occurs approximately 2 h after NTG administration [39]. The recordings were performed before and after the first and fifth doses of NTG. To maintain cleanliness and prevent any potential interference, the field was cleaned using 75% ethanol between tests. For recording and behavioral analysis, CinePlexStudioV3 and CinePlexEditorV3 software from Plexon, USA were utilized. The following parameters were recorded: travel distance, travel time, total grooming time, cage climbing times, and head scratching of the mice. It is known that during painful events, natural behaviors such as locomotor activity and exploration may be reduced in rodents [40]. Cage climbing times, which refers to the number of mice 's front foot climbing the wall of the box, reflects exploration behavior. Grooming, both facial and body, is a natural behavior in rodents, and increased grooming, particularly in a specific area such as the head in migraine models, can indicate increased discomfort. These spontaneous behaviors have been evaluated in preclinical studies as indirect markers of distressing experiences. Head scratching, specifically unilateral or bilateral forepaw scratching of the area innervated by the V1 branch of the trigeminal nerve (including the scalp and periorbital area), is considered a manifestation of spontaneous pain behavior in mice [41]. To avoid the potential effects of filament stimulation on behavior, the mice used in this test were separated from the von Frey filament (VFF) group. Additionally, all investigators involved in the experiment were blinded to the treatment groups.

#### Sample collection

The TNC tissues were collected from the mice in two parts of the experiment. The collected tissues would be used for western blot (WB), immunofluorescence (IF), and enzyme-linked immunosorbent assay (ELISA). Additionally, serum samples were collected for cytokine detection using Luminex liquid suspension chip in the second part of the experimental mice.

On the day of sample collection, between 8:00 and 18:00, the mice in each group were sacrificed under deep anesthesia using 1% pentobarbital sodium. Blood samples were collected from a vein near the eyes of the mice and placed in Eppendorf tubes. After 20 min of undisturbed incubation, the samples were centrifuged for 10 min at 3000 rpm and 4 °C. The resulting serum was divided evenly and stored at -80 °C for subsequent analysis. After cardiac perfusion with PBS or 4% paraformaldehyde (PFA), the effluence became clear, and the liver was stopped after complete blood loss. The neck was quickly severed, and the brain tissue of the mice was removed using tweezers. The TNC tissue was isolated and transferred into a cryogenic storage tube, which was rapidly frozen with liquid nitrogen and stored at -80 °C. Additionally, the TNC tissues of mice injected with 4% PFA were also removed. To prepare the tissue for further analysis of IF, a gradient dehydration process was performed. The tissue was successively transferred to PFA solutions containing 15% and 30% sucrose. Once the tissue sank to the bottom, it was embedded in an optimal cutting temperature compound (OCT) embedding agent and stored at -80 °C. Throughout the entire procedure, an icebox was used to maintain a low temperature, and the brain tissue was carefully handled to ensure the integrity of the dural membrane.

#### Western blot (WB)

The TNC tissues were homogenized in radioimmunoprecipitation (RIPA) lysis buffer (P0013B, Beyotime) using an electric homogenizer and supplemented with a mixture of phenylmethylsulfonyl fluoride (PMSF, ST506, Beyotime) and protease phosphatase inhibitors (P1045, Beyotime). The homogenate was then incubated at 4 °C for 2 h. After incubation, the homogenate was centrifuged at 16,000 rpm and 4 °C for 20 min, and the resulting supernatant was collected as the whole-cell protein extract. The protein concentration was determined using a bicinchoninic acid (BCA) protein assay kit (P0010, Beyotime). Equal amounts of protein were loaded onto a sodium dodecyl sulfate–polyacrylamide gel electrophoresis (SDS-PAGE) gel and electrophoresed. The separated proteins were then transferred to a polyvinylidene fluoride (PVDF) membrane. Subsequently, the membrane was blocked with 5% fetal bovine serum (BSA) at 37 °C for 2 h. Primary antibodies (Supplementary Material 1) were incubated overnight at 4 °C. The membrane was washed three times with tris-buffered saline Tween-20 buffer (TBST) and incubated with a horseradish peroxidase (HRP)-labeled secondary antibody (Supplementary Material 1) at 30 °C for 1 h. Immunoblotting was performed using a blot test kit (SuperFemto ECL Chemiluminescence Kit, Vazyme, E423-02), and the protein bands were visualized using an imaging system (Tanon-5200, China).

#### Immunofluorescence (IF) staining and data analysis

The TNC tissues embedded in OCT were sliced into 10-μm-thick frozen sections using a low-temperature microtome (Leica, 1950 M). The sections were then sealed with a blocking buffer (10% normal goat serum, 0.5% Triton X100, dissolved in 0.1 M PBS) at room temperature for 2 h. Following this, the slices were incubated overnight at 4 °C with a primary antibody (Supplementary Material 1) diluted in the blocking buffer. After washing with PBST three times, the secondary antibody (Supplementary Material 1) was diluted with a closed buffer and incubated for 1 h at room temperature. Subsequently, the sections were rinsed three times with a sealing buffer and sealed with a quench-resistant sealer containing 4', 6-diaminidine-2-phenylindole (DAPI, P0131, Beyotime). Magnified images (× 20 objectives) were captured under a fluorescence microscope (BX43, Olympus) using CellSens standard software (version 1.18, Olympus). In the negative control, PBS was used instead of the primary antibody, and no positive signal was detected. Approximately 8–10 pieces of the TNC from each mouse were extracted, spanning from the anterior to posterior of each mouse. The damaged brain slices were then removed, and 6 pieces were chosen for analysis per mouse. The bilateral sides of each brain slice underwent analysis. The mean intensity of IF was measured using ImageJ software (version 1.52p, National Institutes of Health).

#### Enzyme-linked immunosorbent assay (ELISA)

ATP, ADP, and AMP levels were measured using ATP, ADP, and AMP ELISA kits, respectively (no. JLC_k6403, JLC_k6401, JLC_k6404, Jingkang Biotechnologies, Shanghai, China). Briefly, after administration of NTG or NS, the TNC regions of mice in each group (*n* = 5) were collected in the same manner. According to the manufacturer’s instructions, the samples were added to the appropriate micro-ELISA strip plate wells and combined with specific antibodies. HRP-conjugated antibodies specific for ATP, ADP, and AMP were then added to each well and incubated. Chromogen Solutions A and B were added for coloring, and a stop solution was added to terminate the reaction. Optical density (OD) was measured spectrophotometrically at a wavelength of 450 nm. ATP, ADP, and AMP concentrations were calculated using a standard curve.

#### Luminex liquid suspension chip detection

The Luminex liquid suspension chip assay was conducted using a Bio-Plex Pro Mouse Cytokine Grp kit (#M60009RDPD) and a Luminex 200 system (Austin, Texas, USA) at Wayen Biotech Shanghai Co., Ltd. (Wayen Biotechnologies, Shanghai, China). The analysis focused on seven immune mediators: IL-1β, IL-4, IL-6, IL-10, TNF-α, IFN-γ, and GM-CSF. Both the serum and TNC areas were examined in each group of mice (*n* = 4).

### Statistical analyses

The sample sizes were determined based on previous experience [42], and all samples were biological replicates. All experiments and data analyses were conducted by investigators who were blinded to the group assignments. The data are presented as the mean ± standard error of the mean (SEM). Statistical analyses were performed using SPSS 25.0 (IBM Analytics, Armonk, NY, USA). The Shapiro–Wilk test was used to assess normal distribution. Statistical differences between two groups were analyzed using the independent sample t-test or Mann–Whitney U test. Multiple comparisons were statistically analyzed using one-way analysis of variance (ANOVA) followed by Dunnett's t-test for post hoc analysis or the Kruskal–Wallis H test with Mann–Whitney U post hoc comparison. Two-way repeated-measures ANOVA was used to analyze the behavioral data. Statistical significance was set at *P* < 0.05.

## Results

### The repeated NTG injections induced hyperalgesia and upregulation of CGRP in TNC

**Fig. 2:**
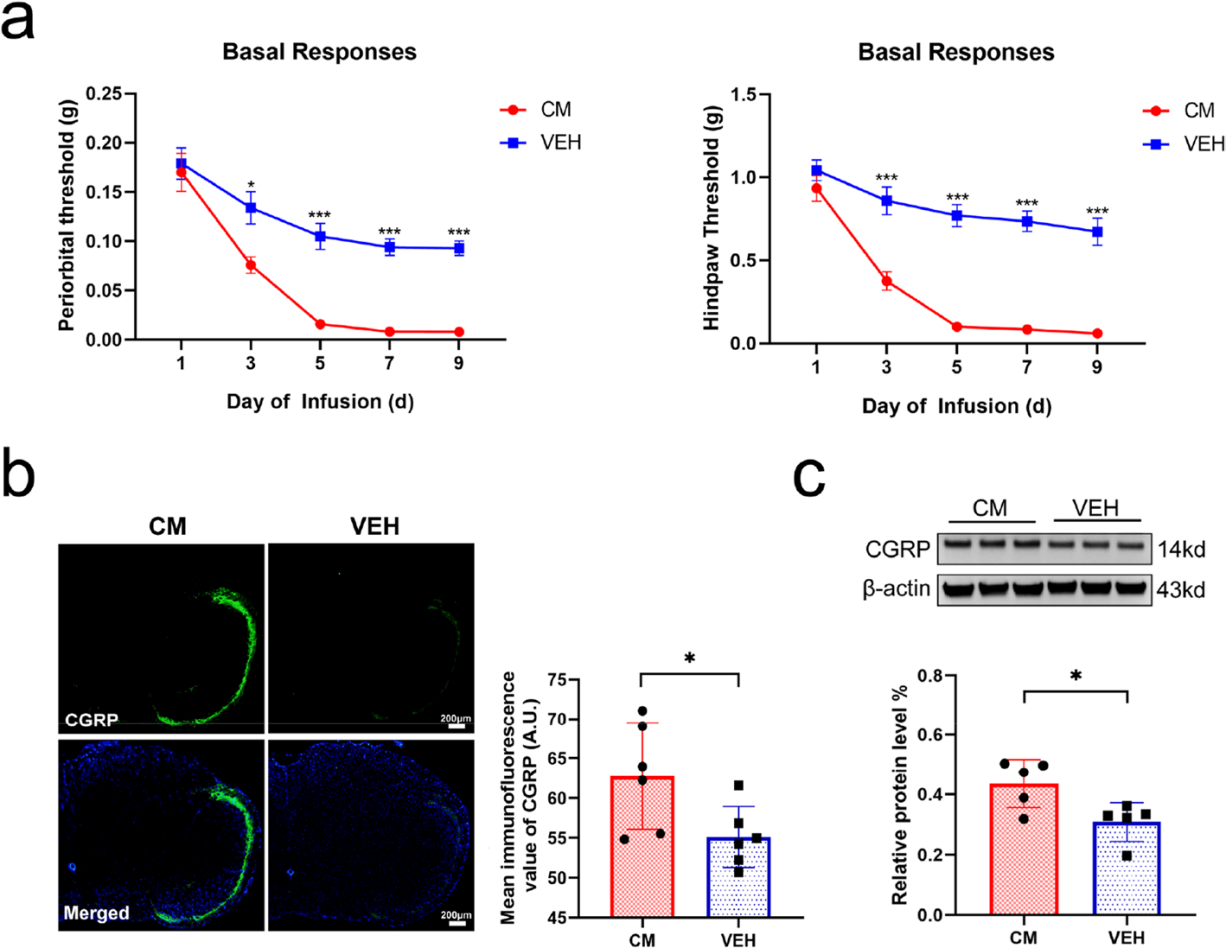
Establishment and evaluation of recurrent NTG-induced CM mouse model. **a** Basal mechanical thresholds in the periorbital region and hindpaw plantar area were assessed in the CM and control (VEH) groups (*n* = 9). **b** Representative photos of calcitonin gene-related peptide (CGRP) immunoreactive staining in the trigeminal nucleus caudalis (TNC) of each group (scale bar = 200 μm) and mean CGRP fluorescence intensity were compared (*n* = 6). **c** Representative western blotting (WB) results for CGRP in each group (*n* = 5). All data were presented as mean ± SEM (standard error of mean). Behavioral data were assessed by two-way repeated-measures ANOVA with post hoc comparison between groups. IF and WB data were assessed by the independent sample t-test or Mann–Whitney U test. (*P < 0.05, **P < 0.01, ***P < 0.001)

The basal mechanical thresholds of the periorbital region and hindpaw plantar area of the mice in both groups exhibited a decreasing trend after administration of NTG or NS, and the mechanical thresholds in the CM group were significantly lower than those in the VEH group on days 3, 5, 7, and 9 (*P* < 0.05), indicating hyperalgesia in the CM group mice (Fig. 2a). In comparison with the VEH group, IF and WB results in the TNC region of mice in the CM group revealed a significant upregulation of CGRP fluorescence intensity and protein expression (*P* < 0.05) (Fig. 2b, c).

### The repeated NTG injections led to a progressive reduction in AMPK activity in TNC

**Fig. 3:**
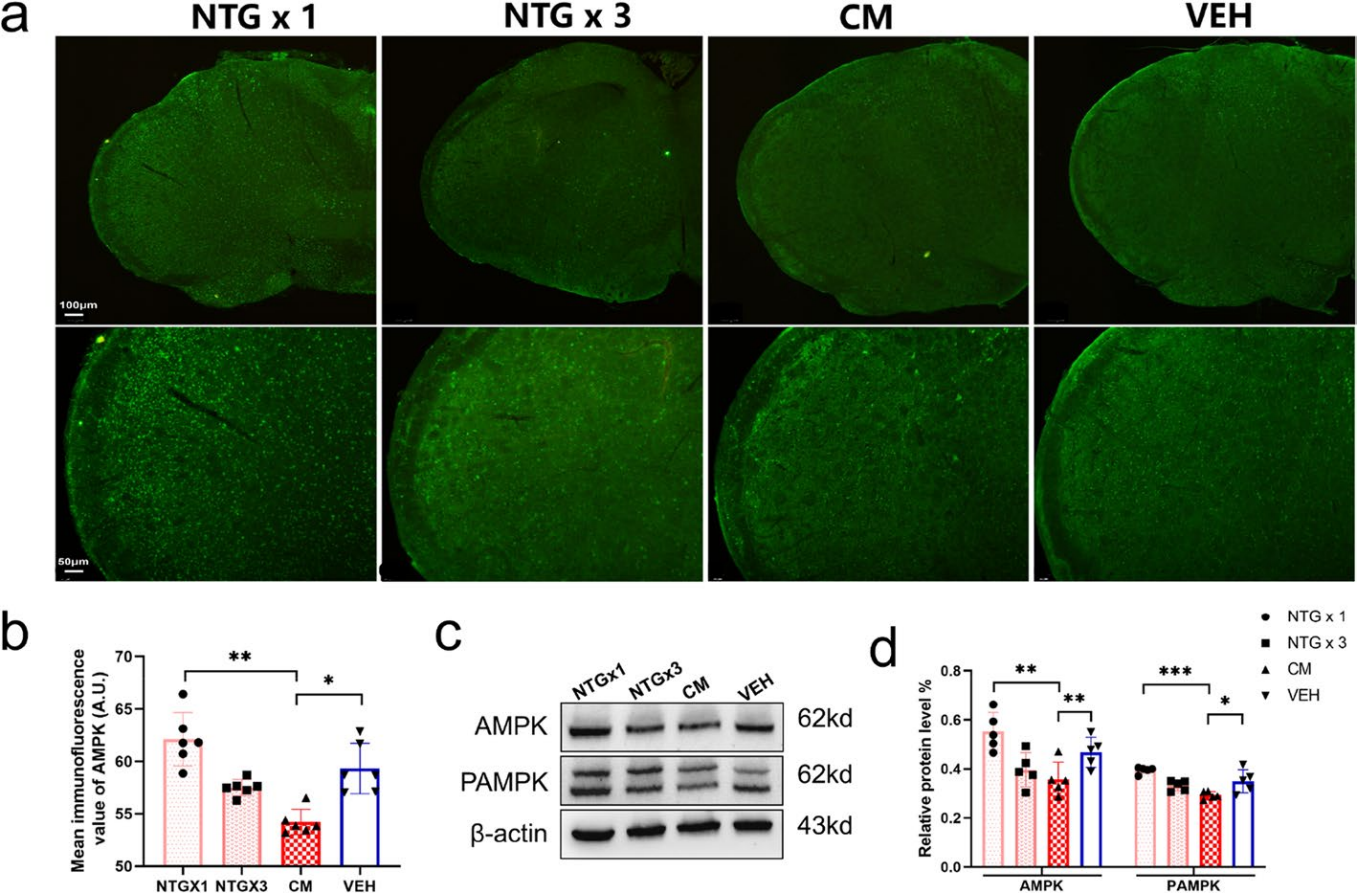
Changes in AMPK activity with an increase in frequency of NTG injection.** a**,** b** Representative images of AMPK immunoreactive staining in the TNC of NTGx1, NTGx3, CM, and VEH groups (upper row scale bar = 100 μm, lower row scale bar = 50 μm). The mean AMPK fluorescence intensity was compared between the groups (*n* = 6). **c**,** d** Representative WB results for AMPK and PAMPK in each group (*n* = 5). All data are presented as the mean ± SEM, and significance was assessed by one-way analysis of variance (ANOVA) with Dunnett’s post hoc test or the Kruskal–Wallis H test with Mann–Whitney U post hoc comparison (*P < 0.05, **P < 0.01, ***P < 0.001)

To investigate the changes in AMPK activity in the NTG-induced CM mouse model during the modeling process, we analyzed the expression changes in NTGx1, NTGx3, CM, and the control group (VEH). IF results showed that the average fluorescence intensity of total AMPK decreased gradually with the increased frequency of NTG administration (Fig. 3a). Additionally, the WB results demonstrated a gradual decrease in the expression levels of total AMPK and p-AMPK proteins (Fig. 3c). Furthermore, the protein expression in the CM group was significantly decreased (*P* < 0.05) compared with that in the VEH group (Fig. 3b, d).

### Contrasting trends in the ADP/ATP ratio and UHRF1 protein expression in the CM model mice

**Fig. 4:**
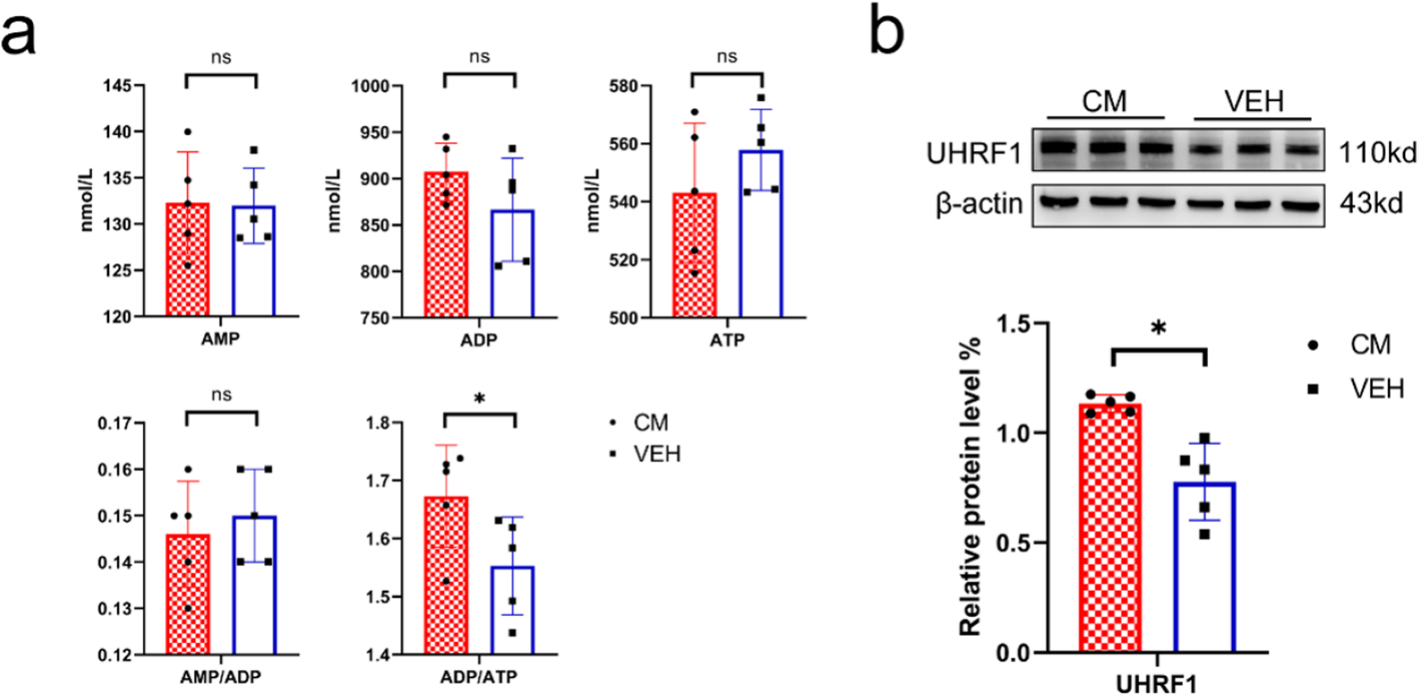
Energy status and UHRF1 protein levels in the TNC region of mice in the CM and VEH groups.** a** Comparison of AMP, ADP, ATP contents and ADP/ATP, and AMP/ADP ratio in the TNC region between the CM and VEH groups (*n* = 5). **b** Representative WB results for UHRF1 in the TNC region of the mice in each group (*n* = 5). All data are presented as the mean ± SEM, and significance was assessed by the independent sample t-test or Mann–Whitney U test between groups (*P < 0.05)

To investigate the cause of this decrease in AMPK activity, we analyzed the positive and negative regulators of AMPK. The concentrations of AMP, ADP, and ATP in the CM group were not significantly different from those in the VEH group, whereas the ADP/ATP ratio was significantly higher in the CM group (*P* < 0.05) (Fig. 4a), which could activate AMPK. However, the WB results for UHRF1 expression showed a significant increase in the CM group (*P* < 0.05) (Fig. 4b), which could inhibit AMPK.

### The activation of AMPK ameliorated hyperalgesia, pain-like behaviors, and impaired mobility in CM model mice

The effects of AICAR and compound C were explored early in the experiment (Supplementary Material 2). An intermediate dose (AICAR 250 μg/g, compound C 10 μg/g) was used for subsequent tests and comparisons.

**Fig. 5:**
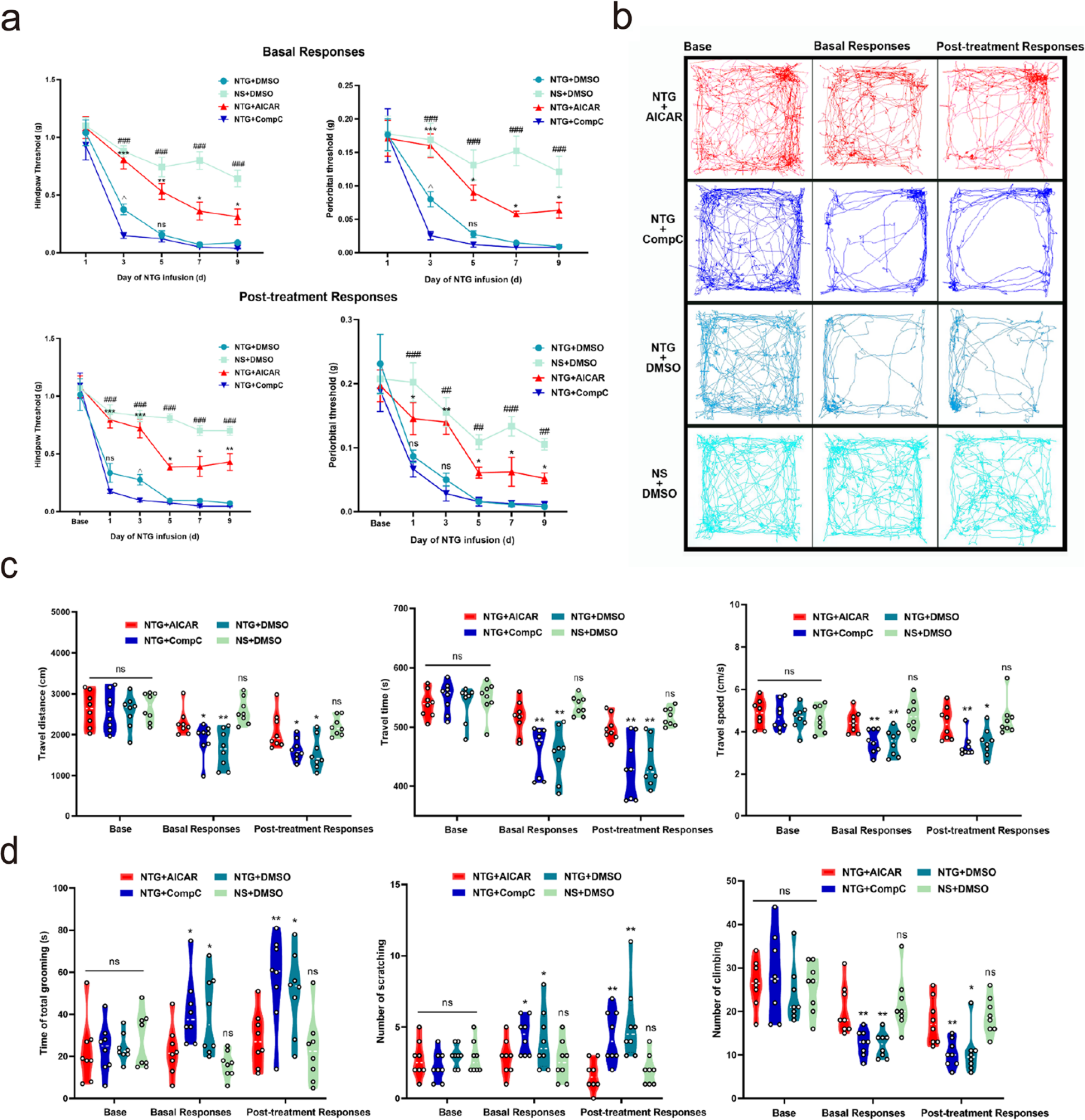
Behavioral results after AMPK intervention.** a** Comparison of basal and post-treatment mechanical pain thresholds of the periorbital region and the hindpaw plantar area in different groups (NTG + AICAR 250 μg/g, NTG + Compound C 10 μg/g, NTG + DMSO, and NS + DMSO, *n* = 9). Significance was assessed by two-way repeated-measures ANOVA with post hoc comparison between groups. (*P < 0.05, **P < 0.01, ***P < 0.001 compared between NTG + AICAR and NTG + DMSO groups, ###*P* < 0.001 compared between NTG + DMSO and NS + DMSO groups, ^*P* < 0.05, ns: not significant compared between NTG + DMSO and NTG + Compound C). **b** Representative trajectory of each group on days 1 and 9. **c**,** d** Comparison of travel distance, time, speed, total grooming time, number of head scratches, and cage climbing in each group. Significance was assessed using one-way ANOVA with Dunnett’s post-hoc test or Kruskal–Wallis H test with Mann–Whitney U post hoc comparison (*P < 0.05, **P < 0.01, compared to NTG + AICAR group, *n* = 8). All data are presented as the mean ± SEM

In terms of mechanical thresholds, multiple NTG injections significantly reduced the basic and post-treatment mechanical pain thresholds of the periorbital region and hindpaw plantar region in the NTG + DMSO group (*P* < 0.05) compared with the NS + DMSO group on days 3, 5, 7, and 9. However, the NTG + AICAR group mitigated the decrease in basal and post-treatment mechanical thresholds (*P* < 0.05) compared to the NTG + DMSO group, suggesting that the AMPK activator AICAR could alleviate pain hyperalgesia induced by NTG. Additionally, the baseline and post-treatment mechanical thresholds of the mice in the NTG + Compound C group decreased quicker than those in the NTG + DMSO group on day 3 (*P* < 0.05) (Fig. 5a).

Consistent with previous findings [43, 44], our study also observed changes in motor behavior in animal models of migraine induced by NTG. Mice in the NTG + DMSO and NTG + Compound C groups displayed a significant decrease in travel distance, time, and speed both before and 2 h after injection, when compared to the NS + DMSO group (*P* < 0.05). In contrast, mice in the NTG + AICAR group exhibited enhanced activity compared to those in the NTG + DMSO and NTG + Compound C groups (*P* < 0.05). However, no significant changes were observed between the AICAR + DMSO and NS + DMSO groups. These findings suggest that AICAR administration improves activity levels in mice. Notably, no significant differences were observed between the NTG + DMSO and NTG + Compound C groups. In contrast to the NTG + AICAR group, the NTG + DMSO and NTG + Compound C groups exhibited a significant increase in pain-like behaviors, such as total grooming and the number of head-scratches, both before and 2 h after injection (*P* < 0.05). However, there was a significant decrease in cage-climbing exploration behavior (*P* < 0.05). Importantly, these pain-like behaviors were not significantly different from those observed in the NS + DMSO group. Therefore, based on these results, it can be inferred that AICAR effectively ameliorates the reduced mobility and pain-like behaviors induced by repeated NTG injections in mice (Fig. 5b-d).

### The activation of AMPK resulted in a decrease in the expression of CGRP, IL-1β, IL-6, and TNF-α, while increasing the expression of IL-4 and IL-10

**Fig. 6:**
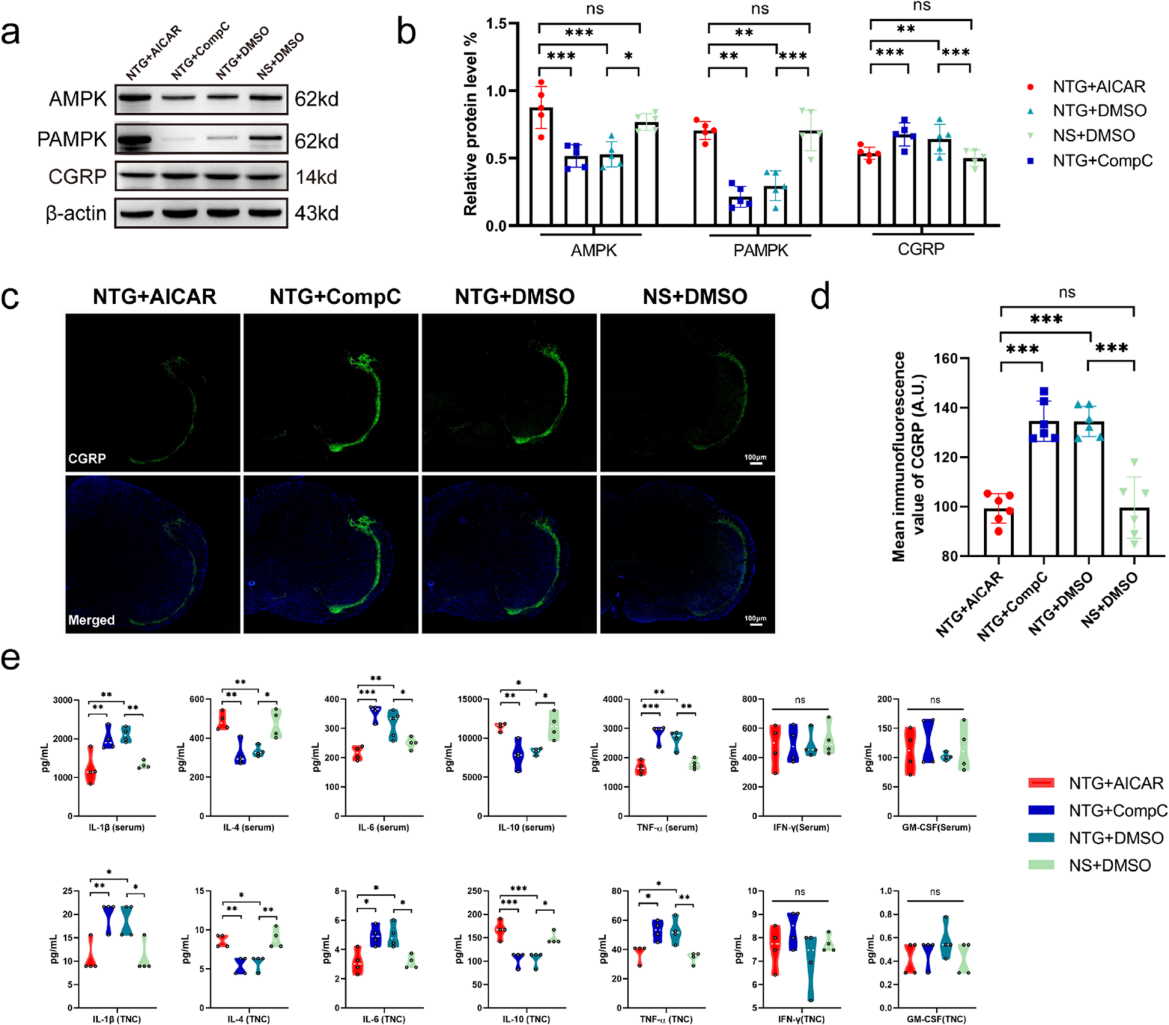
Validation of AMPK expression and CGRP and cytokine responses after activation or inhibition of AMPK.** a**,** b** Representative WB results and comparison of AMPK, PAMPK, and CGRP levels in the TNC region of mice in each group (*n* = 5). **c**,** d** Representative images of AMPK immunoreactive staining (scale bar = 100 μm) and comparison of the mean CGRP fluorescence intensity in each group (*n* = 6). **e** Comparison of the levels of IL-1β, IL-4, IL-6, IL-10, TNF-α, IFN-γ, and GM-CSF in the serum and TNC tissues of mice (*n* = 4). All data are presented as the mean ± SEM, and significance was assessed by one-way ANOVA with Dunnett’s post hoc test or Kruskal–Wallis H test with Mann–Whitney U post hoc comparison (*P < 0.05, **P < 0.01, ***P < 0.001)

We conducted initial verification of AMPK expression in the TNC of mice following intraperitoneal injection of activators and inhibitors. WB analysis revealed that the NTG + AICAR group exhibited significantly higher expression of total AMPK and PAMPK compared to the NTG + Compound C and NTG + DMSO groups. Additionally, the NTG + Compound C group showed a significant reduction in AMPK expression compared to the NS + DMSO group (Fig. 6a, b). Both WB and IF results demonstrated that the NTG + AICAR group displayed significantly lower expression of CGRP compared to the NTG + Compound C and NTG + DMSO groups. However, no significant difference was observed compared to the NS + DMSO group, indicating that AICAR can reduce CGRP expression by activating AMPK. Notably, no significant difference was observed between the NTG + Compound C and NTG + DMSO groups (Fig. 6a-d).

In addition, we collected peripheral serum and TNC tissues from the mice to measure the levels of anti-inflammatory and pro-inflammatory cytokines. Our findings revealed that the levels of IL-1β, IL-6, and TNF-α in the NTG + DMSO group were significantly higher compared to the NS + DMSO group. Conversely, the levels of the anti-inflammatory cytokines IL-4 and IL-10 were significantly decreased in the NTG + DMSO group. In contrast, the NTG + AICAR group exhibited significantly lower levels of IL-1β, IL-6, and TNF-α compared to the NTG + Compound C and NTG + DMSO groups. Furthermore, the levels of IL-4 and IL-10, which are anti-inflammatory cytokines, were significantly increased in the NTG + AICAR group. However, there were no significant differences in the concentrations of IFN-γ and GM-CSF in both serum and TNC among the groups. Additionally, no significant differences in these cytokine levels were observed between the NTG + Compound C and NTG + DMSO groups (Fig. 6e).

### AMPK activated in the TNC region is predominantly localized in microglia

**Fig. 7:**
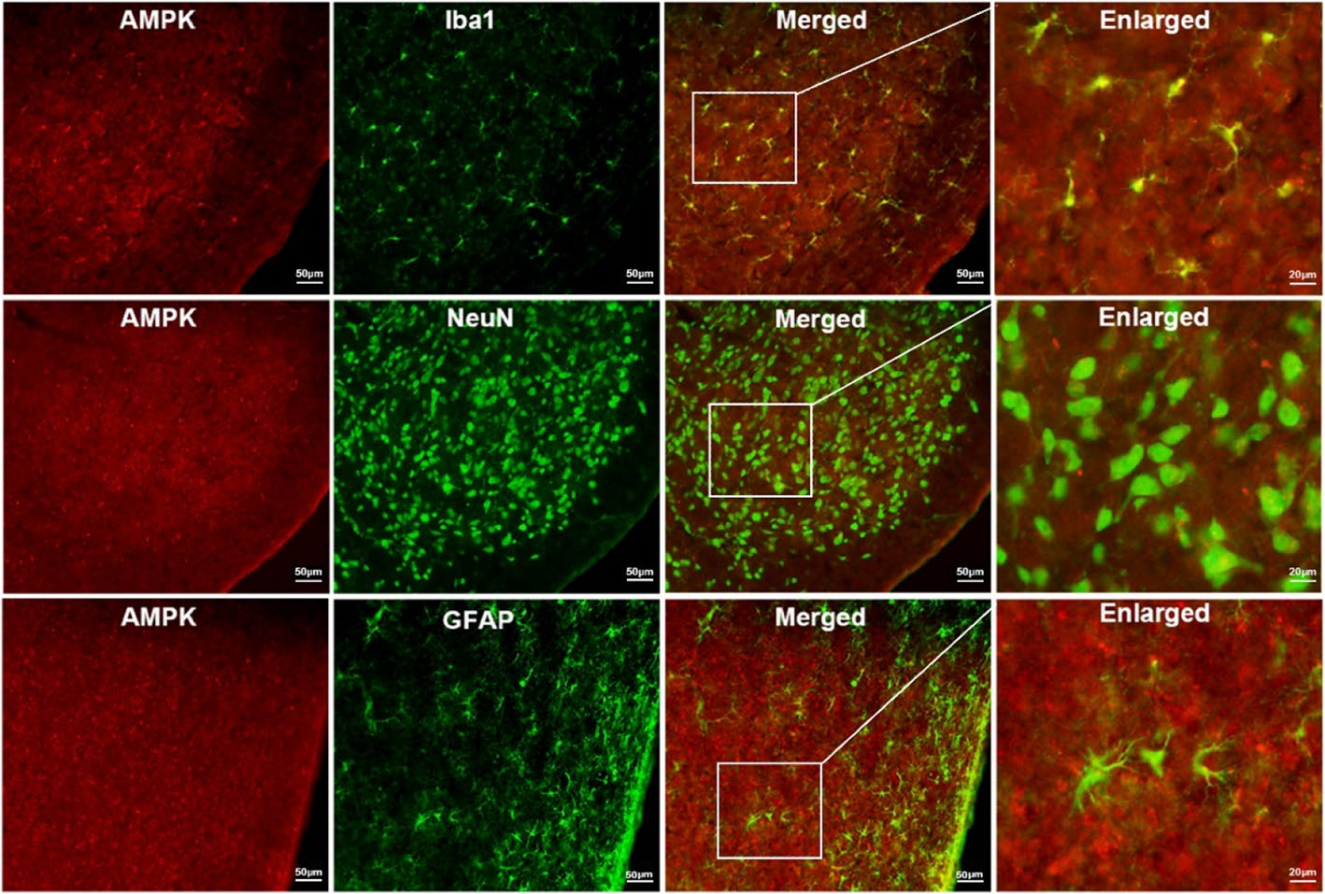
Colocalization staining of AMPK with microglia, neurons, and astrocytes. Double immunofluorescence (IF) labeling of AMPK (red) with Iba-1, NeuN, and GFAP (green) in the TNC after NTG + AICAR administration (scale bar of the first three columns = 50 μm; scale bar of the last column = 20 μm)

To investigate the cellular localization of AMPK in TNC tissue, we performed immunofluorescence staining using specific markers for microglia (Iba1), neurons (NeuN), and astrocytes (GFAP), along with AMPK IF co-staining. The results demonstrated that AMPK in the TNC region was primarily localized in microglia, although there was partial colocalization with neurons and astrocytes as well (Fig. 7).

### AMPK activation leads to M2 polarization of microglia in the TNC region

**Fig. 8:**
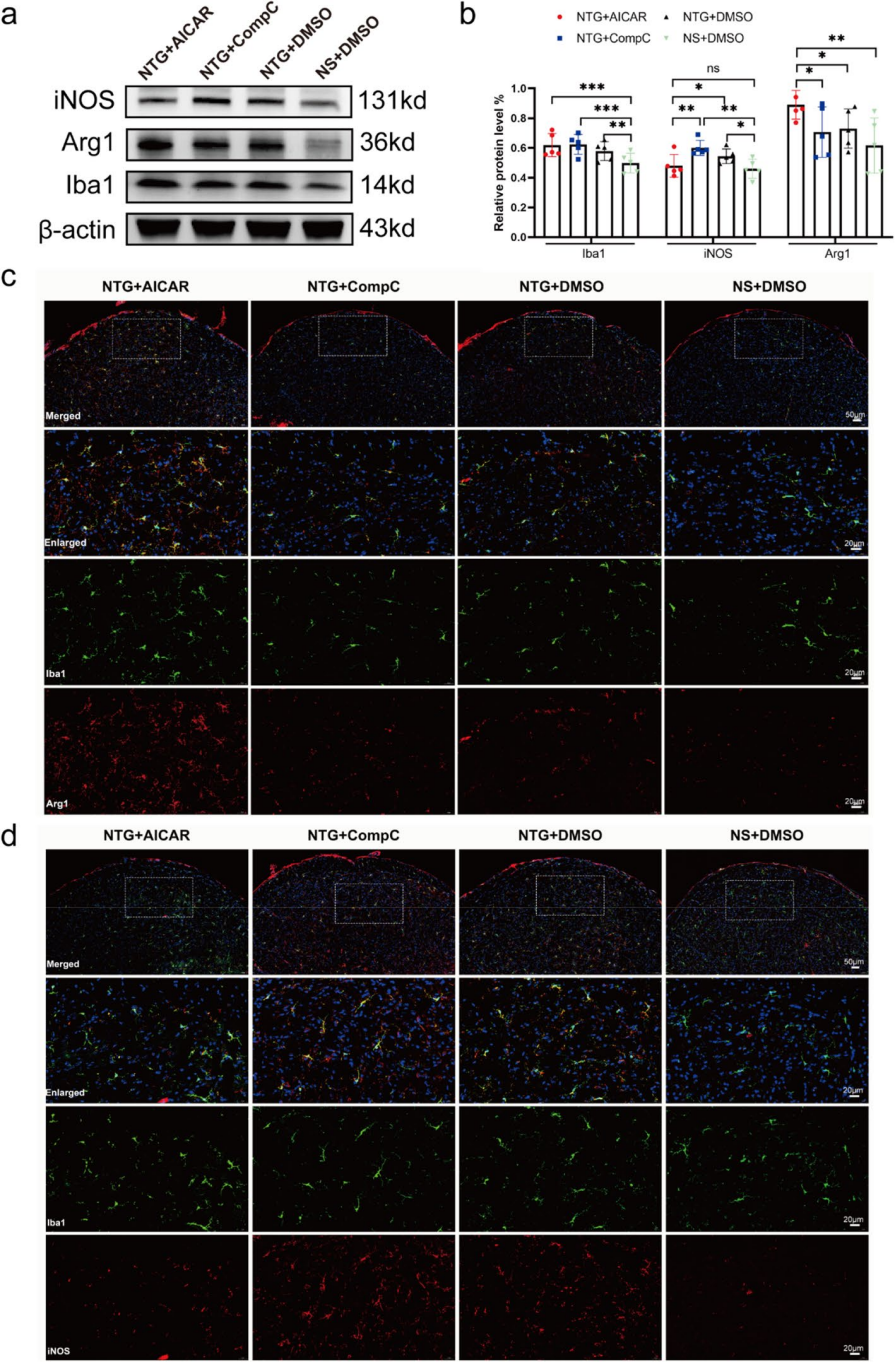
Effect on microglia after activation or inhibition of AMPK expression.** a**,** b** Representative WB results and comparison of Iba1, Arg1, and inducible NO synthase (iNOS) expression in the TNC region of mice in each group (*n* = 5). **c**,** d** Representative images of double IF labeling Iba1 (green) with Arg1 (red) and iNOS (red) (upper row scale bar = 50 μm, the next three rows scale bar = 20 μm). All data are presented as the mean ± SEM, and significance was assessed using one-way ANOVA with Dunnett’s post hoc test or Kruskal–Wallis H test with Mann–Whitney U post hoc comparison (*P < 0.05, **P < 0.01, ***P < 0.001)

Given that AMPK was primarily colocalized with microglia, we proceeded to investigate the impact of AMPK on microglia. To assess microglial polarization, we utilized iNOS + Iba1 and Arg1 + Iba1 markers to label M1 and M2 microglia, respectively. WB analysis revealed a significant increase in Iba1 protein expression in the NTG + AICAR, NTG + DMSO, and NTG + Compound C groups compared to the NS + DMSO group (*P* < 0.05), indicating microglial activation. Moreover, the NTG + AICAR group exhibited significantly higher expression of Arg1 protein compared to the NTG + DMSO and NTG + Compound C groups. Conversely, the expression of iNOS protein was significantly lower in the NTG + AICAR group compared to the NTG + DMSO and NTG + Compound C groups, with statistically significant differences (*P* < 0.05) (Fig. 8a, b). Representative IF staining results for Iba1, Arg1, and iNOS in each group are shown in Figs. 8c, d.

### The activation of AMPK can inhibit the activation of the NF-κB pathway and lead to M2 polarization of microglia

**Fig. 9:**
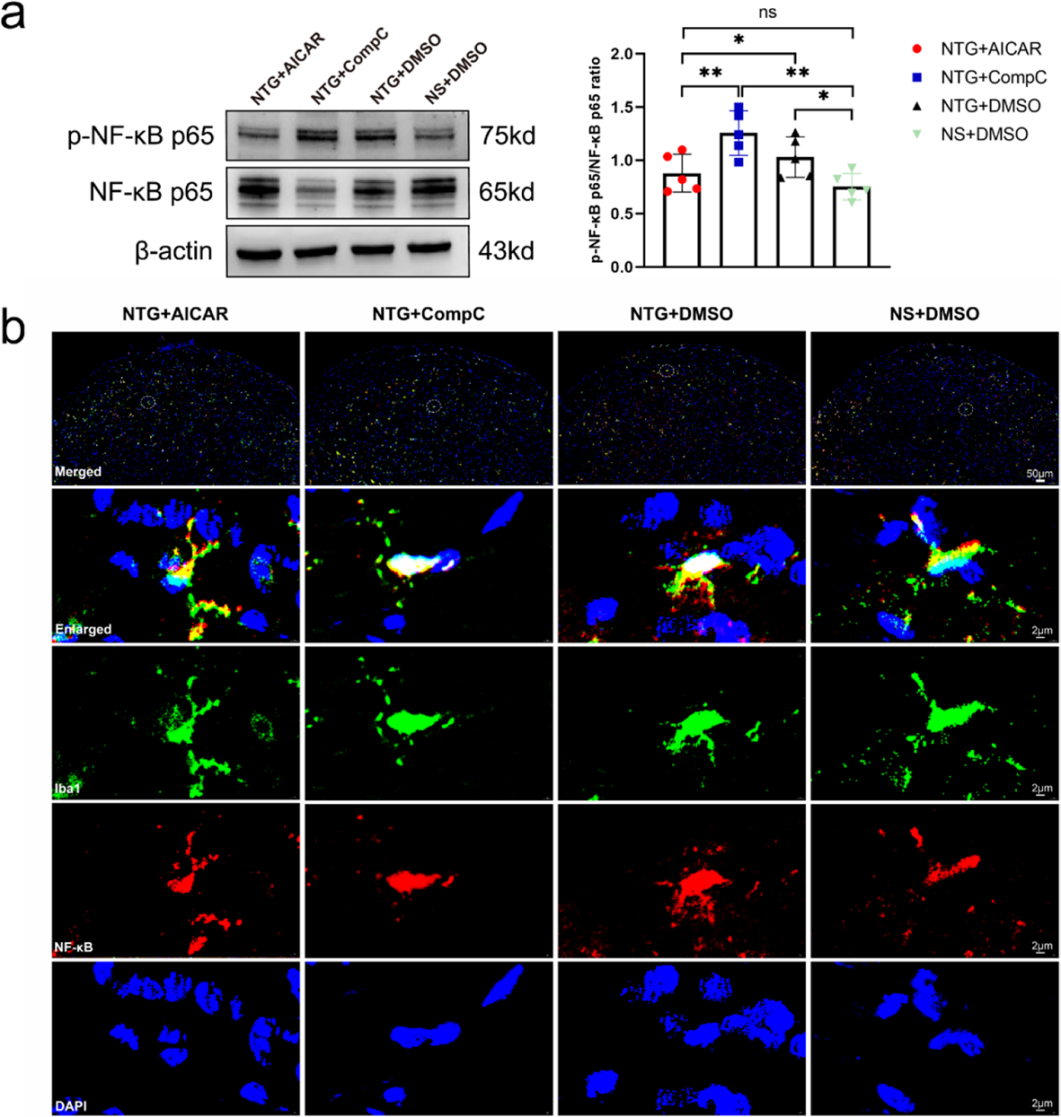
Effect on the NF-κB signaling pathway after activation or inhibition of AMPK expression.** a** Representative WB results and comparison of NF-κB p65 and p-NF-κB p65 in TNC region of mice in each group (*n* = 5). **b** Representative images of IF co-localization staining of NF-κB (red) with Iba1 (green) and DAP1 (blue) (upper row scale bar = 50 μm, the next three rows scale bar = 2 μm). All data are presented as the mean ± SEM, and significance was assessed by one-way ANOVA with Dunnett’s post hoc test or Kruskal–Wallis H test with Mann–Whitney U post hoc comparison (*P < 0.05, **P < 0.01, ***P < 0.001)

To investigate the mechanism underlying microglia M2 polarization, we performed WB analysis to assess the protein expression of NF-κB p65 and p-NF-κB p65 in the TNC region of mice in the NTG + AICAR, NTG + Compound C, NTG + DMSO, and NS + DMSO groups. The results revealed that the ratio of p-NF-κB p65/NF-κB p65 in the NTG + AICAR group was significantly lower compared to the NTG + Compound C and NTG + DMSO groups. However, there was no significant difference compared to the NS + DMSO group, indicating that AICAR could inhibit the activation of the NF-κB pathway (Fig. 9a). Additionally, IF co-localization staining was employed to examine the activation of the NF-κB pathway in microglia. The results demonstrated that the number of cells with plasma-stained NF-κB was higher in the AICAR group, while the NTG + Compound C and NTG + DMSO groups exhibited more nuclear-stained NF-κB (Fig. 9b).

## Discussion

Our study provides the first evidence that the expression of AMPK in the TNC region gradually decreases during the chronic process of migraine in repeated NTG-induced CM model mice. Additionally, the increased expression of UHRF1 may contribute to the inhibition of AMPK activation caused by an elevated ADP/ATP ratio. The activation of AMPK was found to alleviate hyperalgesia and pain-like behaviors while inhibiting CGRP synthesis in mice. Furthermore, our study highlights the colocalization of AMPK with microglia and demonstrates that AMPK activation in the TNC region can suppress the NF-kB pathway, leading to M2 polarization of microglia. This polarization shift results in the reduction of pro-inflammatory cytokines such as IL-1β, IL-6, and TNF-α, and the promotion of anti-inflammatory cytokines IL-4 and IL-10, thereby alleviating central sensitization. These findings may enhance understanding of the molecular mechanisms underlying CM and provide potential targets for therapeutic interventions.

The activation of AMPK pathway primarily involves an increase in intracellular AMP/ATP or ADP/ATP ratios [45]. Upstream kinases, such as liver kinase B1 (LKB1) and Ca2 + /calmodulin-dependent protein kinase β (CAMKK2), mediate AMPK activation in response to energy stress, hormonal and stress conditions, nutritional starvation, exercise, hypoxia [23], and ROS levels [46]. In the context of migraine, most triggers activate AMPK, which acts as a feedback regulatory mechanism to restore brain energy homeostasis and promote headache recovery. In our study, we observed that AMPK was activated and increased at an early stage after a single injection of NTG in the model mice. However, the difference was not significant, possibly due to the timing of sample collection on the second day after injection rather than 2 h post-NTG administration. As the number of injections increased, AMPK activity gradually decreased, indicating an imbalance in feedback regulation and confirming abnormal energy regulation in the process of CM. Furthermore, we discovered an increased expression of UHRF1, a protein involved in DNA methylation, DNA damage repair, and cell proliferation, in CM mice [47, 48]. UHRF1 negatively regulates AMPK activity by facilitating the interaction between AMPK and PP2A phosphatase [24], rather than through upstream kinases LKB1 and CAMKK2. Consequently, despite an increase in the ADP/ATP ratio in CM model mice, AMPK was inhibited rather than activated, possibly due to the elevated expression of UHRF1. However, further validation is required to confirm this mechanism.

CGRP is a crucial component in both migraine and neuropathic pain and is primarily produced in the central and peripheral nervous systems. It is synthesized in the spinal cord (SC), dorsal root ganglion (DRG), and trigeminal ganglion (TG) [49]. Following synthesis in TG neurons, CGRP is stored in dense core vesicles within the cell body, as well as in peripheral and central neurites and terminals [50]. Upon activation of trigeminal neurons, CGRP is released from both afferent and efferent fibers, as well as neuronal cell bodies. Consequently, CGRP is considered a biomarker for migraines [51]. The CALCA gene, responsible for CGRP synthesis, is regulated by the proximal cyclic AMP (cAMP) response element and distal cell-specific enhancer. Its transcription is activated by stimulation of cAMP and mitogen-activated protein kinase (MAPK) signaling pathways [52]. Resveratrol, an AMPK activator, has been shown to reduce CGRP expression in the spinal trigeminal nucleus (STN) and inhibit microglia and astrocyte activation, thereby alleviating trigeminal neuralgia [53]. In obese rats, a high-fat diet-induced enhancement of neuropathic pain is associated with decreased AMPK activity in the SC and DRG, leading to increased CGRP expression independent of nerve injury [54]. This study is the first to demonstrate that AMPK activation reduces CGRP expression in a recurrent NTG-induced chronic migraine mouse model. Furthermore, phosphorylation of AMPK has been shown to inhibit the c-Jun N-terminal kinase/p38 mitogen-activated protein kinase (JNK/p38 MAPK) pathway in a mouse model of intracerebral hemorrhage, resulting in reduced expression of inflammatory factors such as TNF-α and IL-1β in activated microglia [55]. Therefore, AMPK activation may impact the transcription and synthesis of the CGRP gene by inhibiting the activation of the MAPK signaling pathway, ultimately leading to a reduction in CGRP expression.

Neurogenic inflammation plays a crucial role in the development and occurrence of migraines. Glial cells, particularly microglia and astrocytes, play a significant role in the development of central sensitization in response to injury. CGRP activates glial CGRP receptors, leading to the activation of intracellular secondary messenger cAMP through protein kinase A (PKA). This process contributes to the establishment and maintenance of central sensitization [56]. In the NTG-induced chronic migraine model, microglial activation in the TNC mediates the release of inflammatory factors and neurotransmitters through various purinergic receptors, such as P2X4 [57], P2X7 [58], P2Y12 [59], and toll-like receptors [60]. Adenosine, a neuromodulator involved in pain transmission, may impact the synaptic transmission of glutamate in the brainstem by modulating intracellular cAMP or AMPK activity in neurons and glia, thereby contributing to headaches [61–63]. Our study demonstrated a significant increase in the expression of Iba1 protein in microglia in the intervention groups compared to the VEH group, indicating microglial activation. Moreover, our findings revealed that AMPK is primarily colocalized with microglia, suggesting that the regulation of microglia is highly influenced by AMPK. These findings underscore the importance of understanding the role of AMPK in modulating immune responses in the brain.

Studies on multiple sclerosis and other neurological diseases have reported that activation of AMPK can inhibit the activation of the NF-κB pathway and promote the polarization of microglia to an M2-like phenotype, resulting in the decrease of pro-inflammatory cytokines IL-1β, IL-6, and TNF-α and the increase of anti-inflammatory cytokines IL-4 and IL-10, thereby reducing neuroinflammation [64–67]. The activation of NF-κB and increased p65 content in TNC are involved in migraine pathogenesis [68]. AMPK promotes the activity of SIRT1, FOXO, PGC1α, and p53 and inhibits the activity of NF-κB [69, 70]. Our research also found that intraperitoneal injection of AICAR activated AMPK in the TNC region and inhibited the activation of the NF-kB pathway in microglia, resulting in M2 transformation of microglia and corresponding changes in downstream cytokines.

Metformin, which is a type of AMPK activator, has been found to alleviate neuropathic pain in several animal and human studies. Research suggests that metformin may reduce diabetic neuropathic pain by activating the AMPK signaling pathway in DRGs of diabetic rats and possibly by down-regulating NF-κB [71]. Additionally, metformin has been shown to alleviate bortezomib-induced neuropathic pain by inhibiting autophagy in the spinal dorsal horn via regulation of AMPKα2 [72]. Studies have also demonstrated that activating AMPK can alleviate post-surgery pain in mice resulting from incisions [73]. Furthermore, a retrospective study found that metformin reduced pain in diabetic patients with lumbar radiculopathy [74]. There is also evidence suggesting that metformin can reduce mitochondrial dysfunction in fibroblasts of fibromyalgia patients by activating AMPK [75]. While these studies have demonstrated the potential of AMPK activators, including metformin, in treating neuropathic pain, there is currently limited research on the use of AMPK activators in migraine. However, our study provides a foundation for exploring the application of AMPK activators in migraine treatment. In the future, further validation of the effects of metformin on migraine models and clinical studies on its treatment for migraine patients could be conducted to expand our understanding and potential therapeutic options for migraines.

Our study had several limitations that should be acknowledged. Firstly, we only used male mice in our experiments and did not include female controls. This limits the generalizability of our findings to both sexes. Future studies should include female mice to better understand potential sex differences in the effects of AMPK activation on migraines. Secondly, the intraperitoneal mode of drug administration may have influenced metabolic processes throughout the body, rather than solely in the TNC brain region. While we observed AMPK expression in the TNC region following intraperitoneal injection, it is important to consider the systemic effects of the administered drugs. Additionally, the pilot experiment revealed that stereotactic injection of drugs into the TNC area resulted in potential damage and abnormal agitation, and even mortality in mice. Therefore, we opted for intraperitoneal administration for practical reasons. However, this route of administration may not specifically target the TNC region. Furthermore, AMPK activation has diverse effects on various processes, including protein, lipid, and sugar metabolism, autophagy, and mitochondrial homeostasis. While our study focused on the effects of AMPK activation on microglia and cytokine expression, there may be other mechanisms involved in the pathogenesis of migraines that were not investigated. Despite these limitations, the effectiveness of intraperitoneal administration of AMPK agonists in our study shows potential for clinical use in migraine patients. However, further research is necessary to gain a deeper understanding of the underlying mechanisms involved in the development of migraine models and their translation to clinical applications in patients.

## Conclusions

**Fig. 10:**
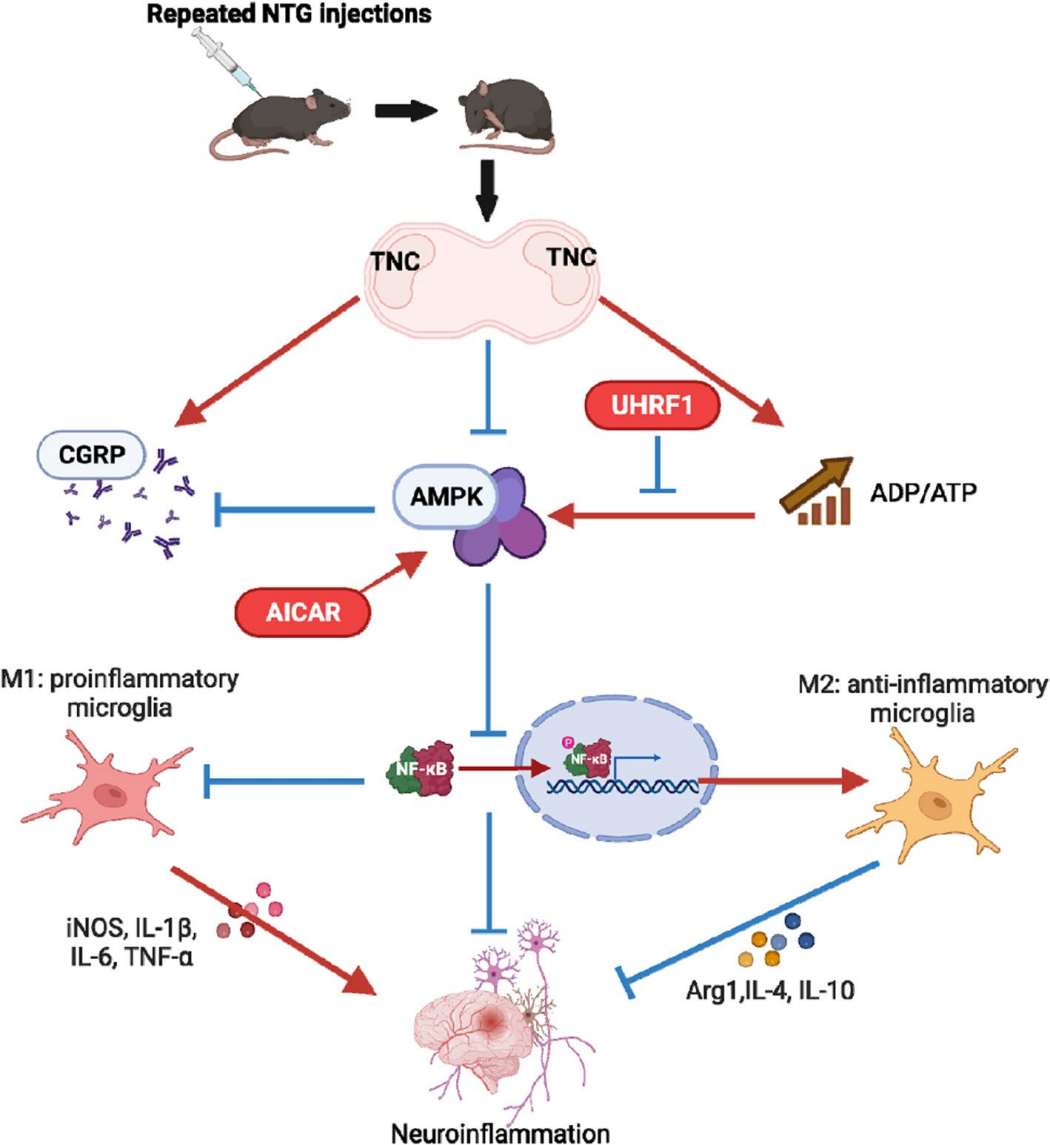
Mechanism of AMPK involvement in central sensitization of chronic migraine. The expression of AMPK decreases in the TNC region of the NTG-induced CM mouse model, and the increased expression of UHRF1 may block the abnormal activation of AMPK caused by an increase in the ADP/ATP ratio. Activation of AMPK can inhibit the synthesis of CGRP and inhibit the activation of the NF-kB pathway, leading to microglia M2-type polarization, thereby inhibiting the secretion of pro-inflammatory cytokines IL1-β, IL-6, and TNF-α and promoting the secretion of anti-inflammatory cytokines IL-4 and IL-10 to alleviate central sensitization

In summary, our study provides compelling evidence for the critical involvement of AMPK in the central sensitization of CM, particularly in the context of abnormal energy metabolism. Activation of AMPK leads to a decrease in neuroinflammation in NTG-induced CM mice through the M2 polarization of microglia, as illustrated in Fig. 10. This discovery establishes a clear connection between “abnormal energy metabolism” and “neurogenic inflammation,” offering researchers a promising new target for the treatment of CM. The modulation of AMPK activity could potentially provide therapeutic benefits in managing CM by addressing the underlying mechanisms of central sensitization.

### Supplementary Information

**Additional file 1.** Antibodies used for western blot analysis and immunofluorescence staining.

**Additional file 2.** Comparison of basal mechanical pain thresholds after intervention with different AMPK activator and inhibitor doses.

## Data Availability

The data used in this article are available from the corresponding author upon reasonable request.

## Abbreviations

AMPK: AMP-activated protein kinase
Arg1: Arginase 1
CM: Chronic migraine
NTG: Nitroglycerin
TNC: Trigeminal nucleus caudalis
AICAR: 5-Aminoimidazole-4-carboxyamide ribonucleoside
CGRP: Calcitonin gene-related peptide
UHRF1: Ubiquitin-like with plant homeodomain and RING finger domains 1
GBD: Global Burden of Disease
YLDs: Years lived with disability
EM: Episodic migraine
ROS: Reactive oxygen species
iNOS: Inducible NO synthase
NS: Normal saline
i.p.: Intraperitoneally
DMSO: Dimethyl sulfoxide
vFF: Von Frey filaments
PBS: Phosphate-buffered saline
PFA: Paraformaldehyde
OCT: Optimum cutting temperature compound
RIPA: Radioimmunoprecipitation
PMSF: Phenylmethyl sulfonyl fluoride
BCA: Bicinchoninic acid
SDS-PAGE: Sodium dodecyl sulfate-polyacrylamide gel electrophoresis
PVDF: Polyvinylidene fluoride
BSA: Bovine serum
TBST: Tris buffered saline Tween-20 buffer
HRP: Horseradish peroxidase
WB: Western blot
IF: Immunofluorescence
ELISA: Enzyme-linked immunosorbent assay
DAPI: 4', 6-Diaminidine—2-phenylindole
SEM: Standard error of the mean
ANOVA: One-way analysis of variance
LKB1: Liver kinase B1
CAMKK2: Calcium-sensing kinase Ca2 + /calmodulin-dependent protein kinase β
cAMP: Cyclic AMP
SC: Spinal cord
DRG: Dorsal root ganglion
STN: Spinal trigeminal Nucleus
MAPK: Mitogen-activated protein kinase
PKA: Protein kinase A
