# Supplementary material for: AMPK activation attenuates central sensitization in a recurrent nitroglycerin-induced chronic migraine mouse model by promoting microglial M2-type polarization
Source: J Headache Pain. 2024 Mar 8;25(1):29. doi: 10.1186/s10194-024-01739-w (PMC10921743; doi:10.1186/s10194-024-01739-w)
Supplement: Supplementary file 1 — Additional file 1. Antibodies used for western blot analysis and immunofluorescence staining. [file 10194_2024_1739_MOESM1_ESM.docx]

**Supplementary Material 1**

Antibodies used for western blot analysis and immunofluorescence staining

| **Antibody** | **Manufacturer** | **Catalog number** | **Host** | **Dilution** |
| --- | --- | --- | --- | --- |
| For western blot analysis  AMPK  P-AMPK (Thr172)  CGRP  iNOS  Arg1  Iba1  NF-κB p65  NF-κB phospho-p65  β-Actin  Rabbit IgG (HRP)  For immunofluorescence staining  AMPK  NF-KB p65  CGRP  NeuN  Iba1  GFAP  iNOS  Arg1  Alexa Fluor 488 goat anti-rabbit IgG  Alexa Fluor 488 goat anti-chicken IgG  Alexa Fluor 488 donkey anti-goat IgG  Alexa Fluor 594 goat anti-rabbit IgG | Cell Signaling Technology  Cell Signaling Technology  Abcam  Abcam  Abcam  Abcam  Proteintech  Proteintech  Beyotime  Beyotime  Abcam  Abcam  Abcam  Abcam  Abcam  Abcam  Abcam  Abcam  Abcam  Abcam  Abcam  Abcam | 2532  2535  ab139264  ab202417  ab203490  ab178847  80979-1-RR  82335-1-RR  AF5003  A0208  ab2387  ab16502  ab36001  ab134014  ab289874  ab4674  ab178945  ab203490  ab150077  ab150173  ab150129  ab150080 | rabbit  rabbit  rabbit  rabbit  rabbit  rabbit  rabbit  rabbit  rabbit  goat  rabbit  rabbit  goat  chicken  goat  chicken  rabbit  rabbit  goat  goat  donkey  goat | 1:1000  1:1000  1:1000  1:1000  1:1000  1:1000  1:5000  1:2000  1:2000  1:1000  1:500  1:200  1:1000  1:1000  1:400  1:1000  1:500  1:200  1:1000  1:1000  1:1000  1:1000 |
| Alexa Fluor 594 donkey anti-rabbit IgG | Abcam | ab150076 | donkey | 1:1000 |
