# Supplementary material for: AMPK activation attenuates central sensitization in a recurrent nitroglycerin-induced chronic migraine mouse model by promoting microglial M2-type polarization
Source: J Headache Pain. 2024 Mar 8;25(1):29. doi: 10.1186/s10194-024-01739-w (PMC10921743; doi:10.1186/s10194-024-01739-w)
Supplement: Supplementary file 2 — Additional file 2. Comparison of basal mechanical pain thresholds after intervention with different AMPK activator and inhibitor doses. [file 10194_2024_1739_MOESM2_ESM.docx]

**Supplementary Material 2** Comparison of basal mechanical pain thresholds after intervention with different AMPK activator and inhibitor doses


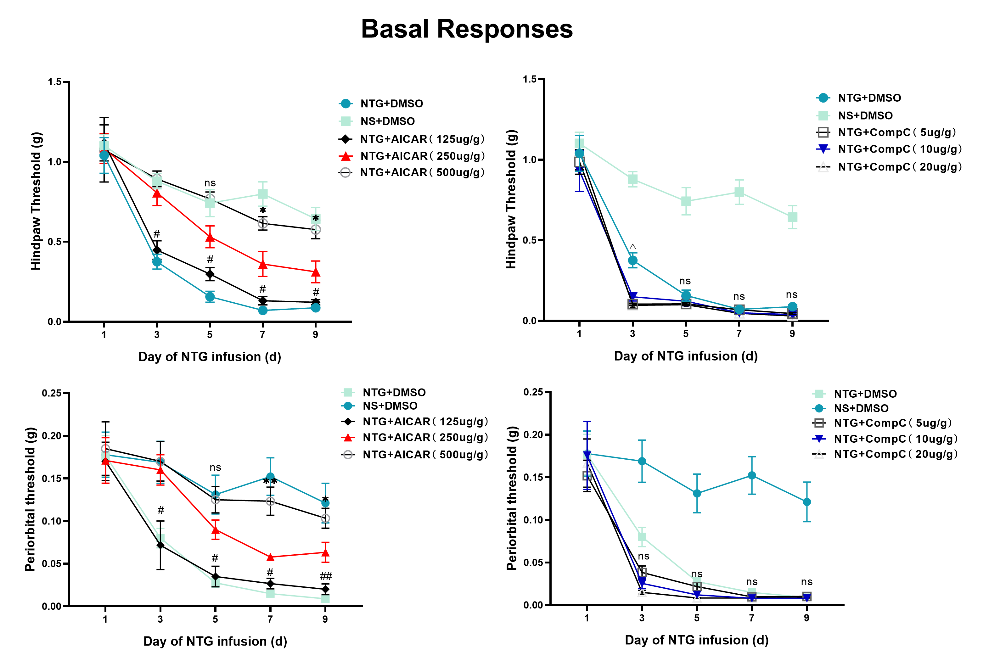


The hindpaw and periorbital pain thresholds showed further improvement with an increase in AICAR dose. However, there was no significant difference between the AICAR 125 µg/g and NTG groups. On day 3, the pain threshold of mice significantly decreased at different doses of compound C, resulting in no significant difference observed in the trend chart. Although the group given AICAR at a dose of 500 µg/g showed positive results, the required dosage was higher and more expensive. Therefore, an intermediate dose (AICAR 250 µg/g, compound C 10 µg/g) was used for subsequent tests and comparisons.

All data are presented as the mean ± SEM. Significance was assessed by two-way repeated-measures ANOVA with post hoc comparison between groups. (*P < 0.05, **P < 0.01 compared between NTG+AICAR 250 µg/g and NTG+AICAR 500 µg/g groups, #P < 0.05, ##P < 0.01 compared between NTG+AICAR 250 µg/g and NTG+AICAR 125 µg/g groups, ^P < 0.05, ns: not significant  compared between NTG + DMSO and NTG + Compound C groups).
